# Supplementary material for: Reciprocal expression of MADS-box genes and DNA methylation reconfiguration initiate bisexual cones in spruce
Source: Commun Biol. 2024 Jan 19;7:114. doi: 10.1038/s42003-024-05786-6 (PMC10799047; doi:10.1038/s42003-024-05786-6)
Supplement: Supplementary file 2 — Supplementary information [file 42003_2024_5786_MOESM2_ESM.pdf]

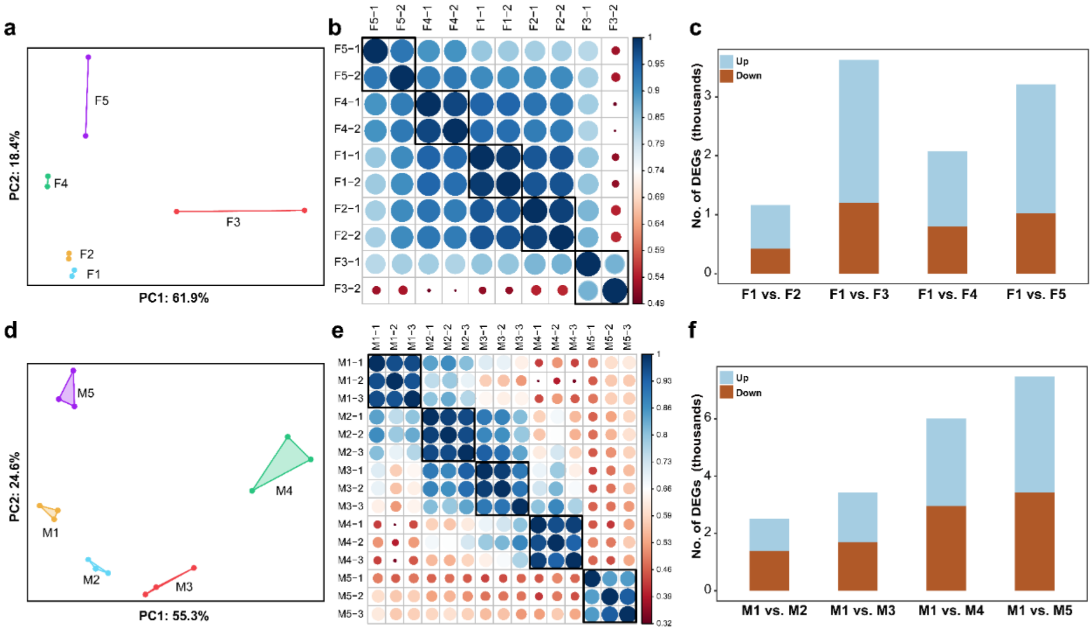

Supplementary Figure 1. Correlation analysis and differential expression analysis of M1-M5 and F1-F5. a-b Principal component analysis (PCA) and Pearson correlation coefficient analysis among F1, F2, F3, F4 and F5. **c** Number of DEGs of F2, F3, F4, and F5 relative to F1. **d-e** PCA and Pearson correlation coefficient analysis among M1, M2, M3, M4 and M5. **f** Number of DEGs of M2, M3, M4, and M5 relative to M1.

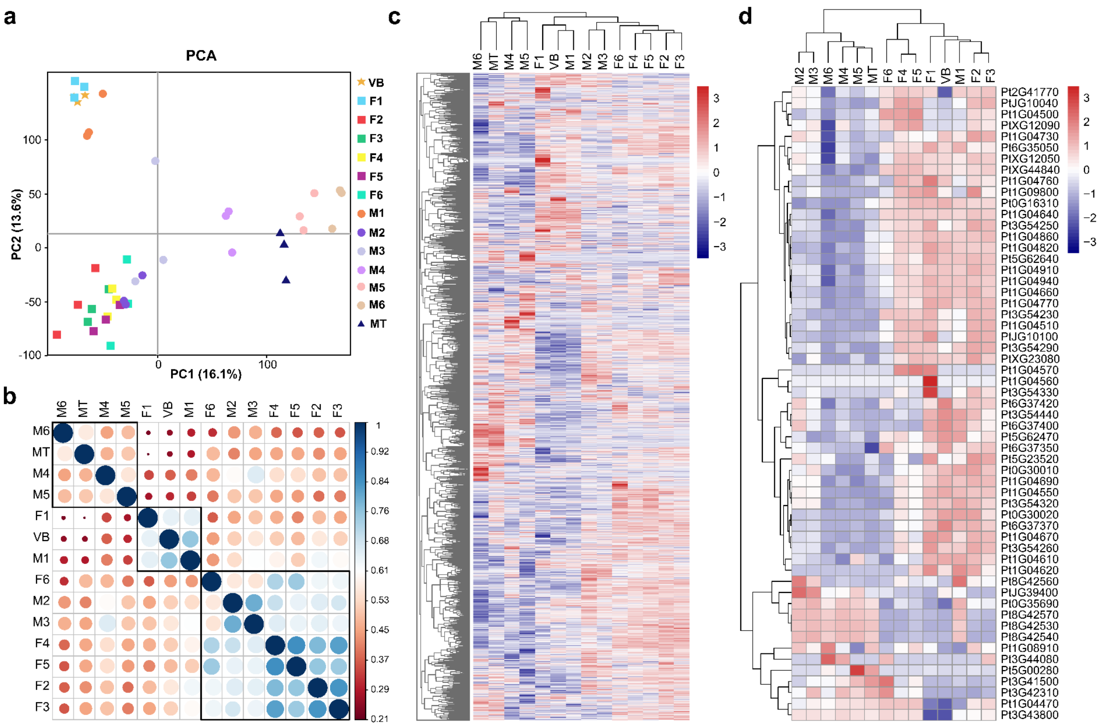

Supplementary Figure 2. Clustering analysis of transcriptome and MADS-box gene expression profiles in *Pinus tabuliformis*. a-c Principal component analysis (PCA), Pearson correlation coefficient and hierarchical clustering analysis of transcriptome expression profiles. **d** Hierarchical clustering analysis of MADS-box gene expression profiles. All the samples analyzed were from Niu et al. (2016). F, female cones; M, male cones; MT, male parts of the bisexual cones; VB, vegetative buds.

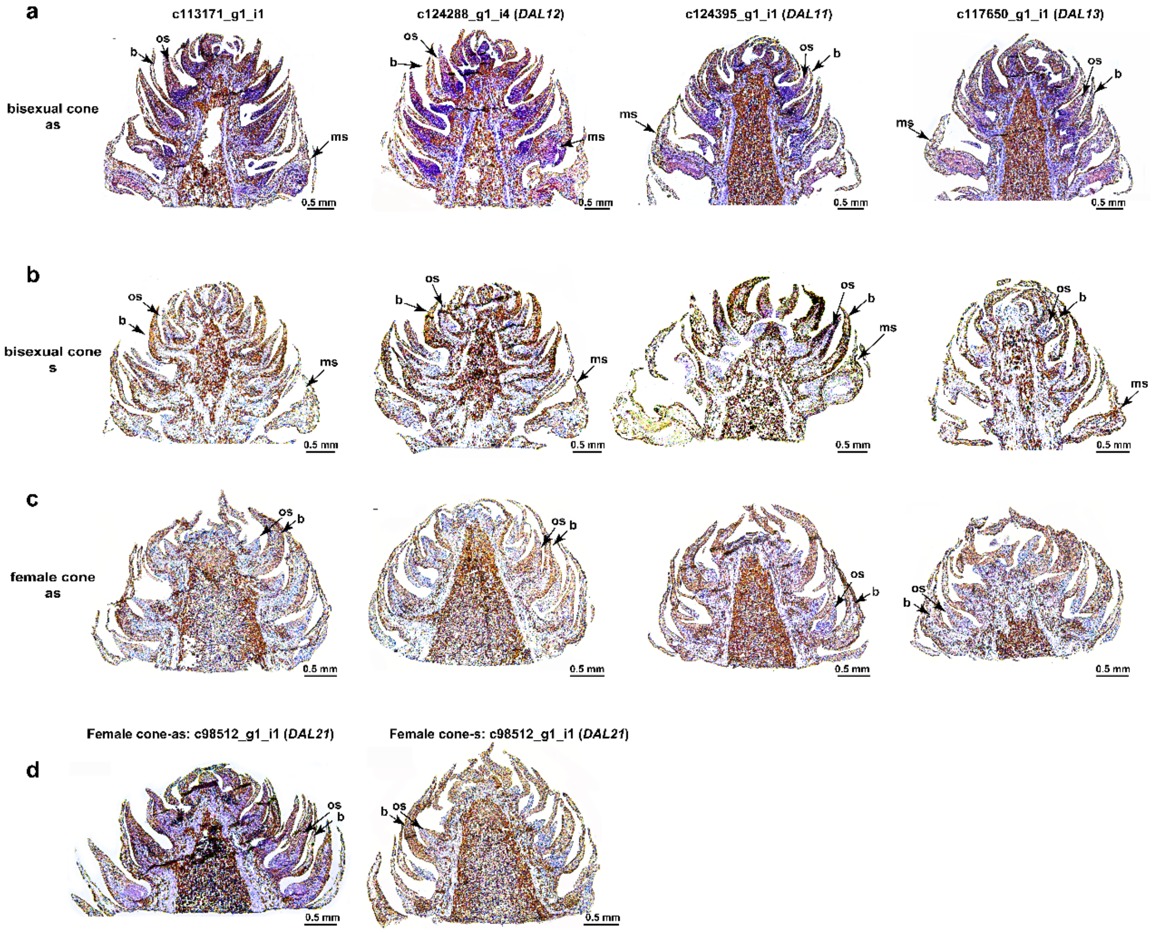

Supplementary Figure 3. *In situ* localization of B-class genes and *PcDAL21*. a-b *In situ* localization of B-class genes in bisexual cones with antisense and sense probes. **c** *In situ* localization of B-class genes in female cones with antisense probes. **d** *In situ* localization of *PcDAL21* in female cones with antisense and sense probes. os, ovuliferous scale; b, bract; ms, microsporophyll. All bisexual and female cones used in *in situ* localization were collected on 7 April, 2019.

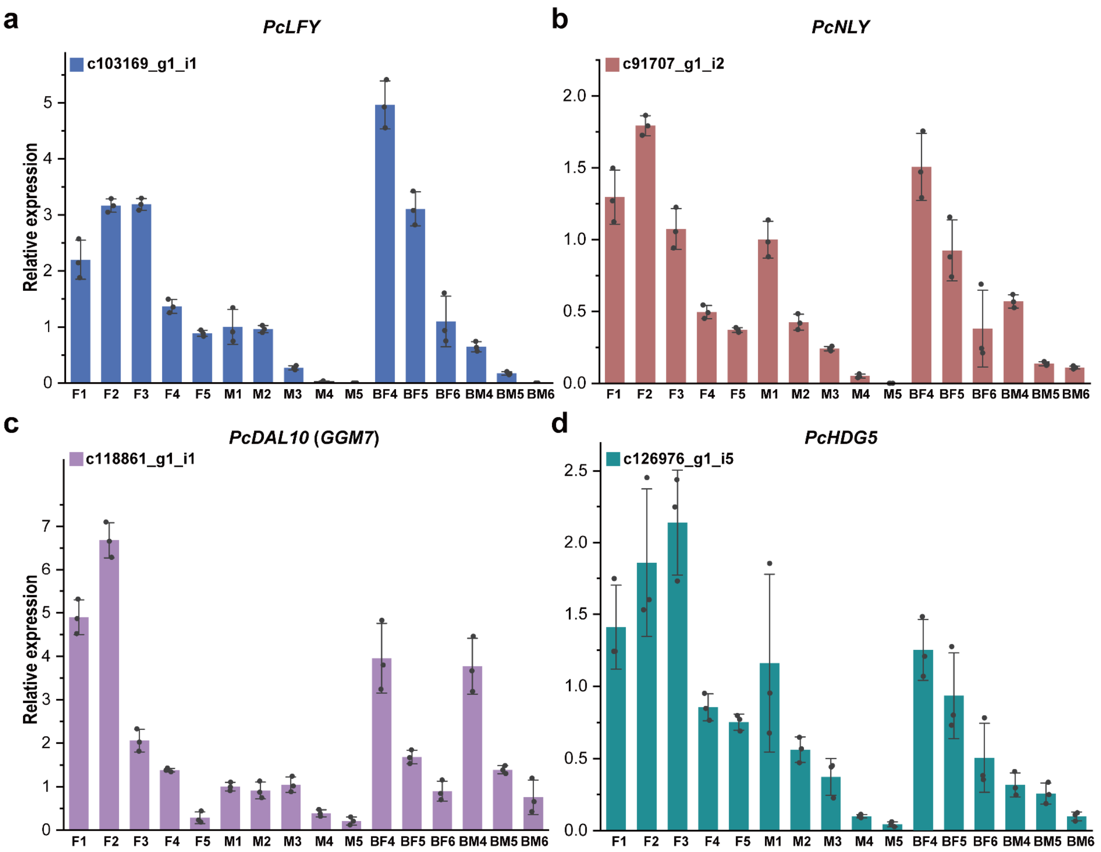

Supplementary Figure 4. QRT-PCR analysis of *PcLFY* (a), *PcNLY* (b), *PcDAL10* (c), and *PcHDG5* (d). The histograms show the mean ± SD (standard deviation) (n = 3 biological independent duplications).

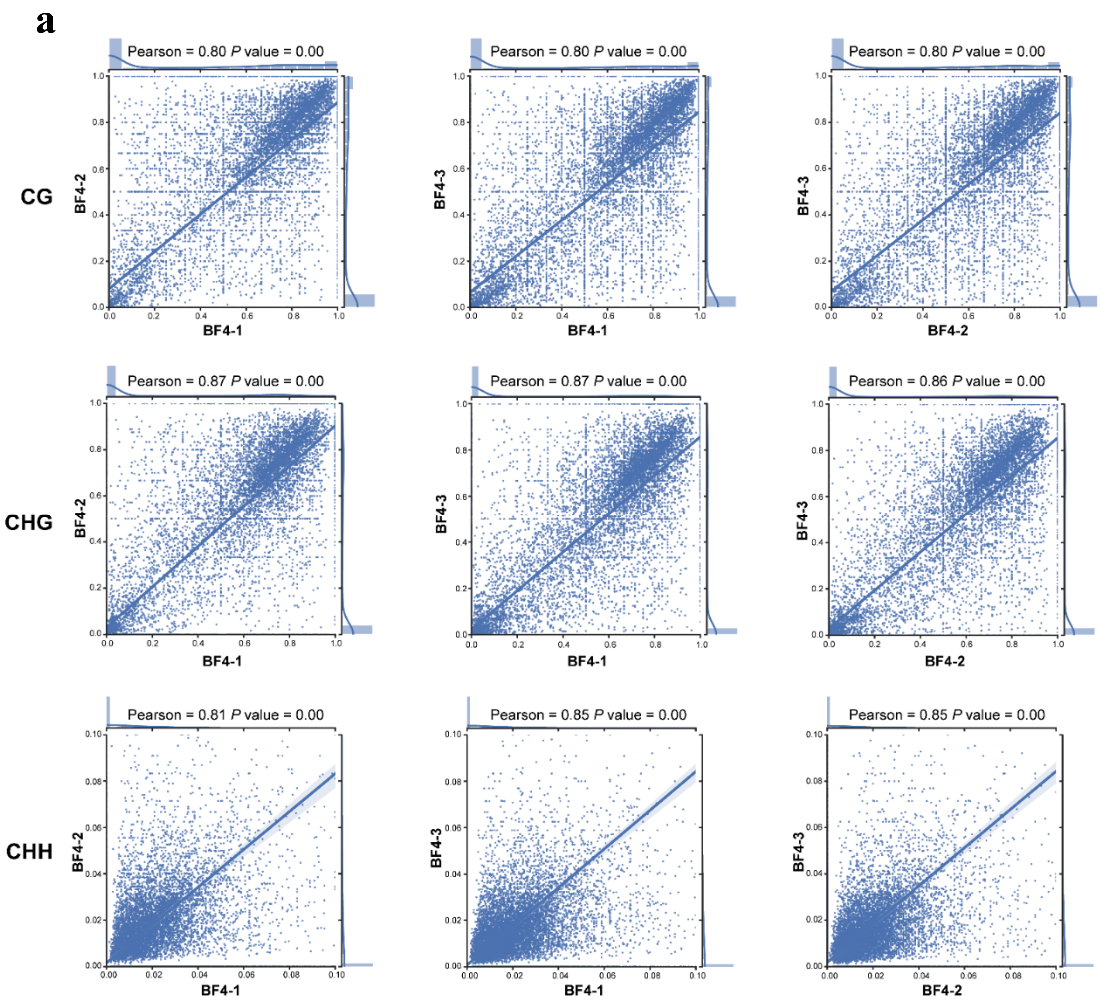

Supplementary Figure 5. DNA methylation correlation analysis among replicates. The Pearson correlation coefficient was estimated based on the methylome data of three biological replicates using Python package Scipy. **a** BF4, **b** BM4, **c** F4, **d** M4.

b

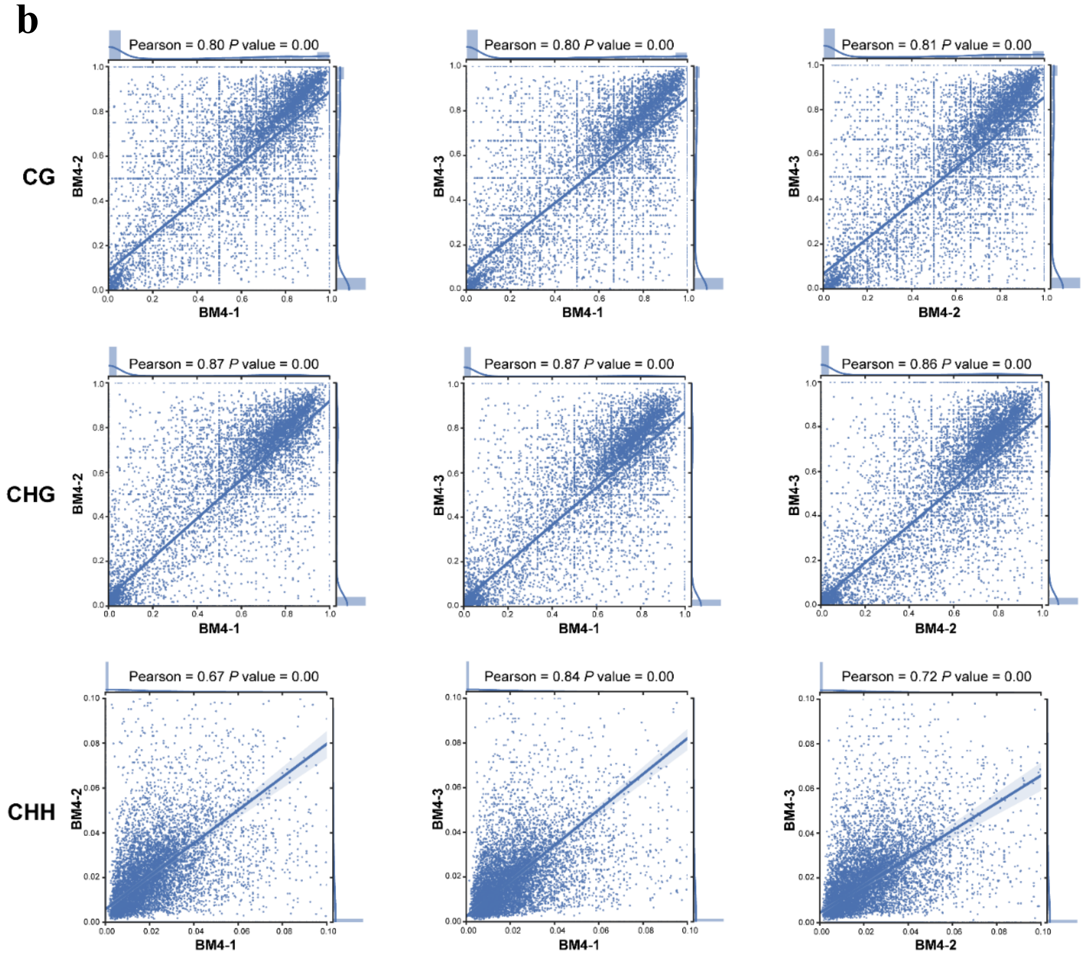

Supplementary Figure 5. DNA methylation correlation analysis among replicates. The Pearson correlation coefficient was estimated based on the methylome data of three biological replicates using Python package Scipy (Continued). **a** BF4, **b** BM4, **c** F4, **d** M4.

c

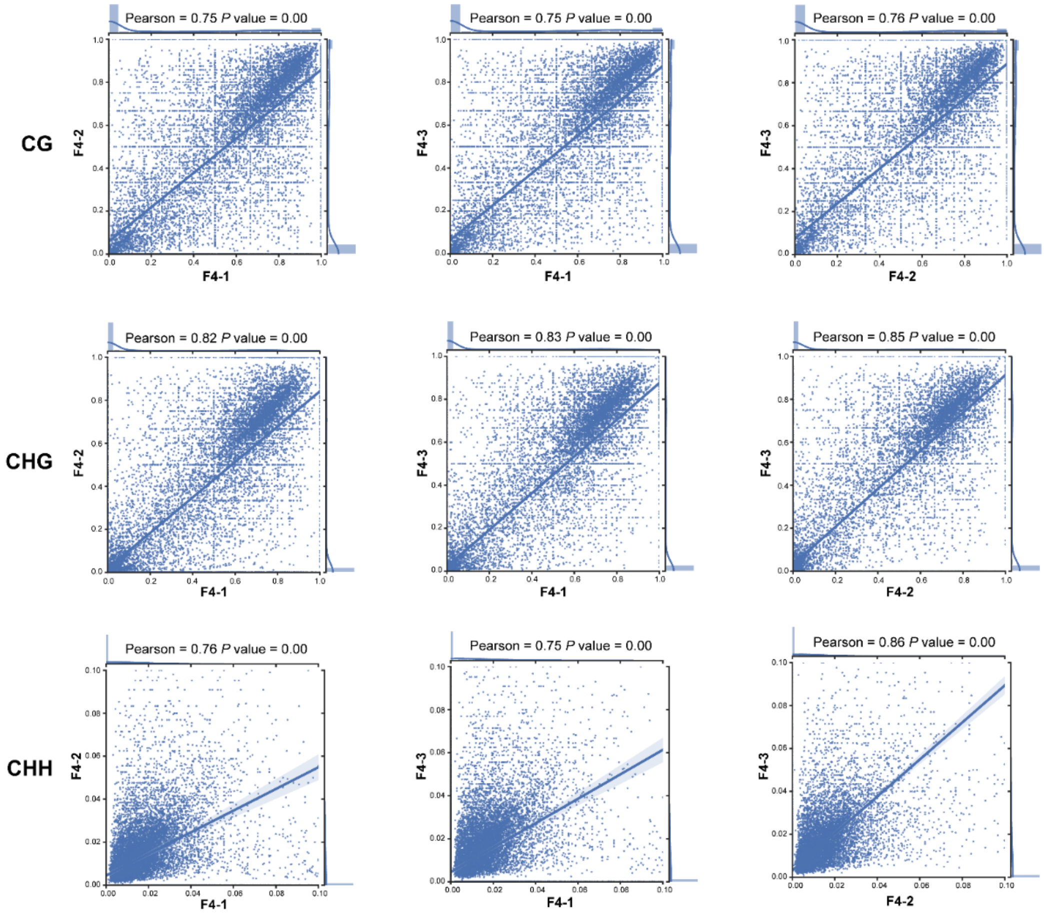

Supplementary Figure 5. DNA methylation correlation analysis among replicates. The Pearson correlation coefficient was estimated based on the methylome data of three biological replicates using Python package Scipy (Continued). **a** BF4, **b** BM4, **c** F4, **d** M4.

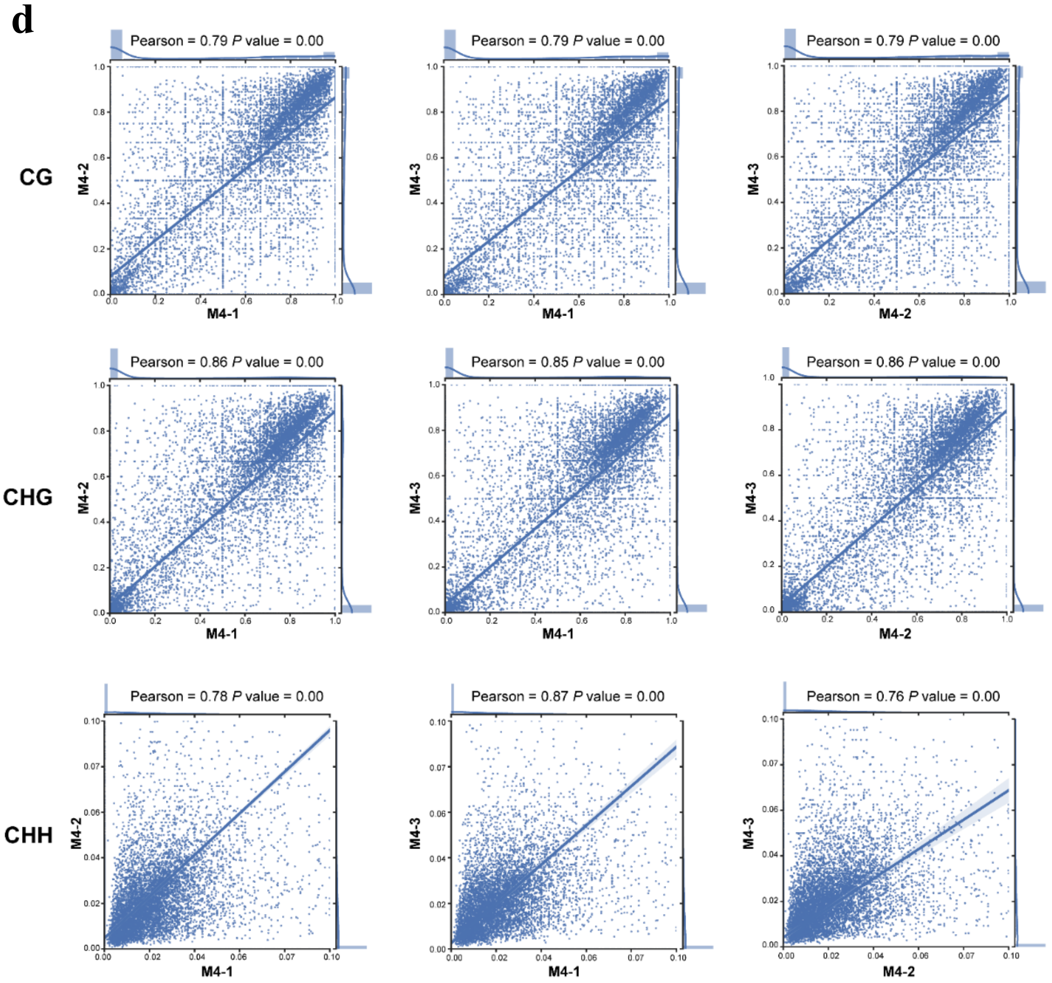

Supplementary Figure 5. DNA methylation correlation analysis among replicates. The Pearson correlation coefficient was estimated based on the methylome data of three biological replicates using Python package Scipy (Continued). **a** BF4, **b** BM4, **c** F4, **d** M4.

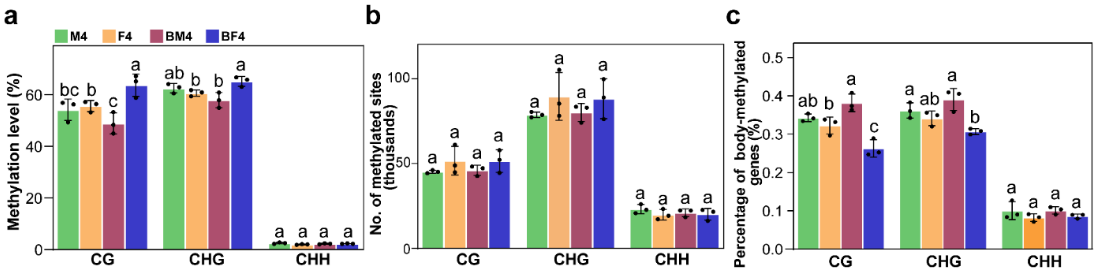

Supplementary Figure 6. DNA methylation characterization of M4, F4, BM4 and BF4. a DNA methylation levels. **b** Number of methylated cytosine sites. **c** Percentage of body-methylated genes. Statistical analysis was conducted using the One-way ANOVA followed by LSD (least significant difference) analysis based on the data from three biological duplications of M4, F4, BM4 and BF4. Different letters denote significant differences ($P < 0.05$). The histograms show the mean ± SD (standard deviation) (n = 3 biological independent duplications). The methylation data of each replicate was individually evaluated.

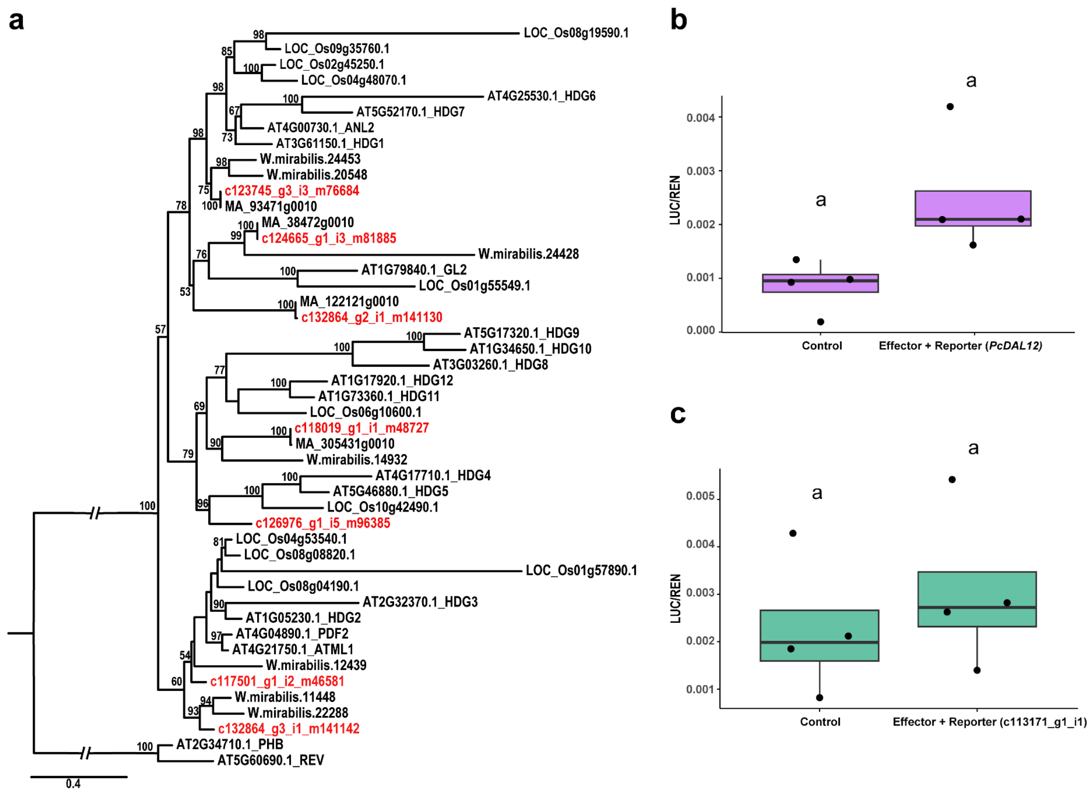

Supplementary Figure 7. Phylogenetic analysis of HD-ZIP IV genes and results of dual-LUC transient expression assays. a ML tree of HD-ZIP IV genes. Quantitative analysis of dual-LUC transient expression assays of the promoter activity of (**b**) *PcDAL12* and (**c**) c113171_g1_i1. Box plot lines represent, from bottom to top, 25th percentile, median and 75th percentile of the data dispersion. Statistical analysis was conducted using the two tailed Student's *t*-test (n = 4 biological independent duplications) and different letters denote significant differences at *P* value < 0.05.

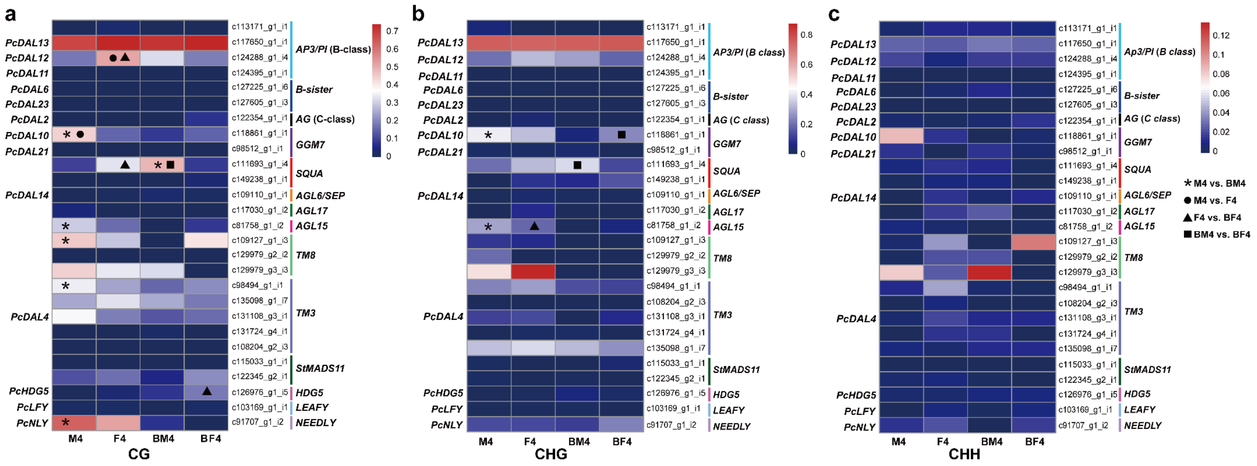

Supplementary Figure 8. CG, CHG and CHH methylation levels in promoters of MADS-box genes, *PcLFY*, *PcNLY* and *PcHDG5*. **a** CG, **b** CHG, **c** CHH. Statistical analysis was conducted using the chi-square test. Asterisks, dots, triangles and rectangles represent significantly increased methylation levels (*P* value < 0.05) in M4 vs. BM4, M4 vs. F4, F4 vs. BF4, and BM4 vs. BF4, respectively.

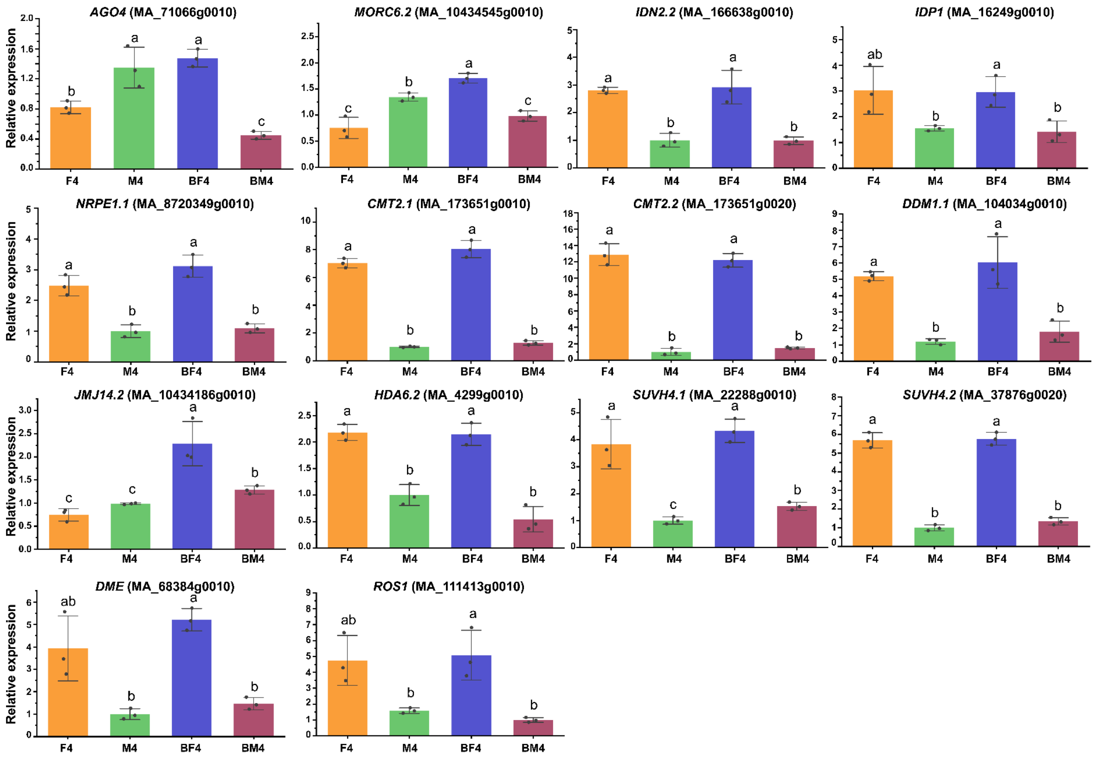

Supplementary Figure 9. qRT–PCR analysis of DEGs involved in *de novo* methylation, maintenance methylation and demethylation processes in F4 vs. BF4 and BM4 vs. BF4. Statistical analysis was conducted using the two tailed Student's *t*-test. Different letters denote significant differences at P value < 0.05. The histograms show the mean ± SD (standard deviation) (n = 3 biological independent duplications).

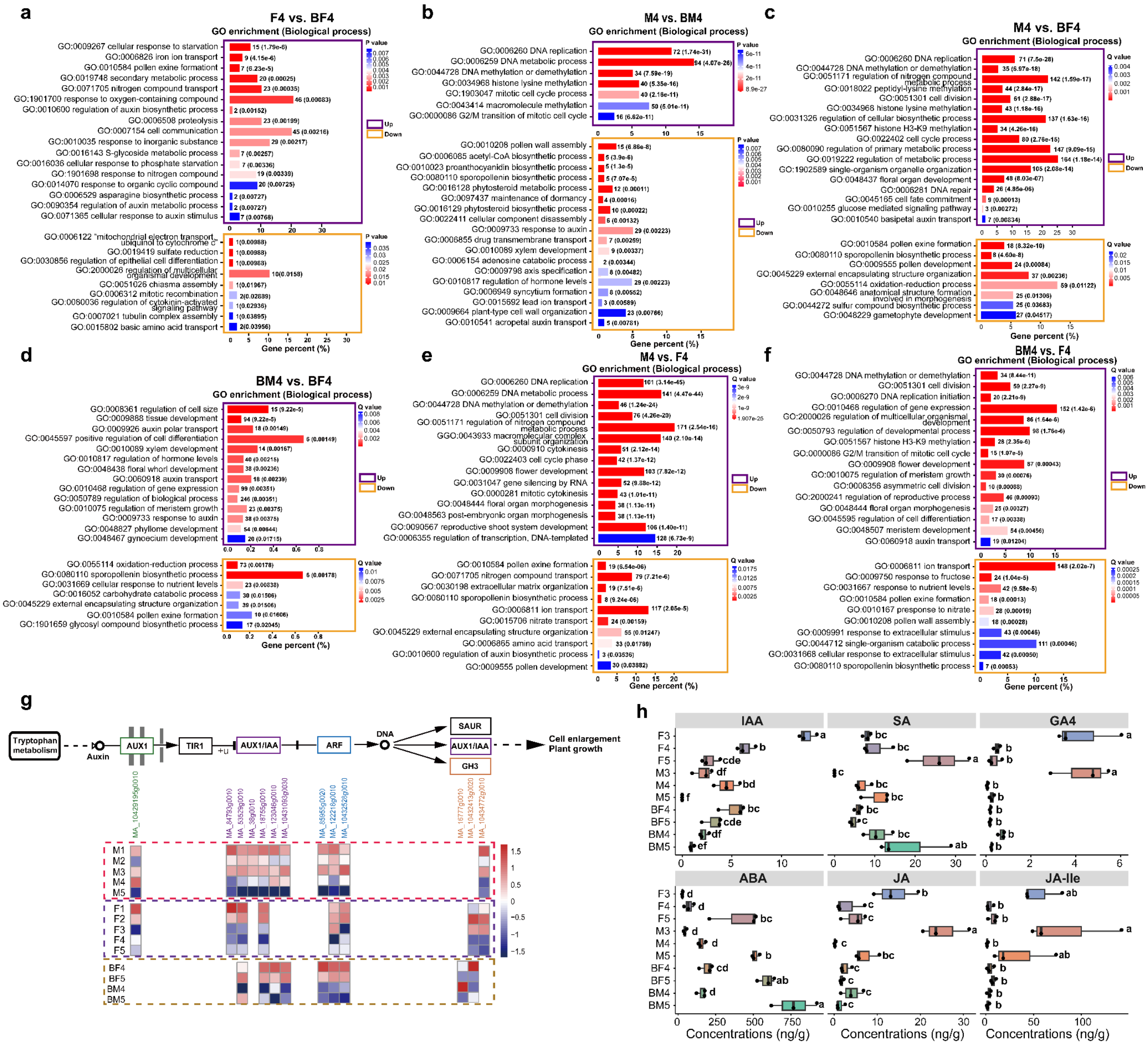

Supplementary Figure 10. GO and KEGG enrichment of DEGs and statistics of hormone contents. Enriched GO terms of DEGs in (**a**) F4 vs. BF4, (**b**) M4 vs. BM4, (**c**) M4 vs. BF4, (**d**) BM4 vs. BF4, (**e**) M4 vs. F4, and (**f**) BM4 vs. F4. (**g**) Expression pattern of DEGs related to the auxin signal transduction pathway identified in M1 vs. M5 (red dotted box), F1 vs. F5 (purple dotted box), and BM4 vs. BF4 (brown dotted box). **h** Statistical box plots of the content of six plant hormones. Box plot lines represent, from left to right, 25th percentile, median and 75th percentile of the data dispersion. Statistical analysis was conducted using the One-way ANOVA followed

by LSD (least significant difference) analysis (n = 3 biological independent duplications), and different letters denote significant differences (*P* < 0.05). Due to the absence of GA1 and GA7 in all samples, as well as the low concentrations of GA3 detected only in BF4 and M5, no statistical analysis was conducted for these hormones. IAA, indole-3-acetic acid; SA, salicylic acid; GA4, gibberellin A4; ABA, abscisic acid; JA, jasmonic acid; JA-Ile, jasmonic acid-isoleucine.

Supplementary Table 1. Germination rates of pollen grains.

	**No. of germinated pollen grains**	**No. of ungerminated pollen grains**	**Sum**	**Germination rate (%)**	***P* value (chi-square test)**
M5	151	150	301	50.16	0.44
BM5	143	163	306	46.73	

Supplementary Table 2. Information of 12 methylomes used in this study.

**Tissue**	**Code**	**Collection date**	**SRA accession numbers**	**No. of raw reads**	**No. of clean reads**	**Conversion Rate (%)**
Male cone	M4-1	07 April 2019	SRR24957455	119,299,529	112,656,436	98.3
	M4-2	07 April 2019	SRR24957454	122,287,389	116,238,153	97.7
	M4-3	07 April 2019	SRR24957451	139,503,413	120,467,890	98.2
Female cone	F4-1	07 April 2019	SRR24957450	161,572,789	132,158,629	98.2
	F4-2	07 April 2019	SRR24957449	162,693,731	145,681,283	98.3
	F4-3	07 April 2019	SRR24957448	142,245,506	129,283,033	98.2
Male structure of bisexual cone	BM4-1	07 April 2019	SRR24957447	117,870,198	111,769,192	98.1
	BM4-2	07 April 2019	SRR24957446	145,516,352	136,706,758	97.9
	BM4-3	07 April 2019	SRR24957445	138,625,109	122,343,992	98.2
Female structure of bisexual cone	BF4-1	07 April 2019	SRR24957444	152,652,216	144,693,820	98.2
	BF4-2	07 April 2019	SRR24957453	119,524,510	112,447,312	98.2
	BF4-3	07 April 2019	SRR24957452	143,884,254	127,724,247	98.3

Supplementary Table 3. Predicted *AtHDG5* (*Arabidopsis thaliana*) binding sites located within the promoter of *PcDAL13* gene according to the JASPAR database.

DNA binding site sequence logo	Score	Relative score	Start	End	Strand	Predicted sequence
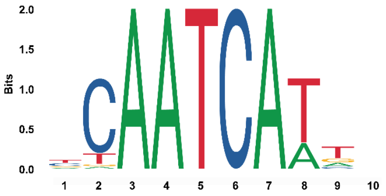	13.14	0.964	2025	2034	-	TCAATCAATA
	10.65	0.914	2771	2780	+	CTAATCATAG
	8.92	0.880	1556	1565	+	TTAATCAAAT
	8.63	0.873	195	204	+	AAAATCATTC
	7.78	0.856	2326	2335	+	ATAATCAAGT
	7.73	0.855	2036	2045	-	ATAATCAAAT

Supplementary Table 4. Information of 37 transcriptomes used in this study.

Tissue	Code	Collection date	SRA accession numbers	No. of raw reads	No. of clean reads
Male cone	M1-1	19 March 2019	SRR24889235	20,719,180	19,676,752
	M1-2	19 March 2019	SRR24889234	25,709,416	24,203,185
	M1-3	19 March 2019	SRR24889223	22,739,209	21,446,619
	M2-1	25 March 2019	SRR24889212	19,898,135	15,656,878
	M2-2	25 March 2019	SRR24889204	18,628,874	15,870,497
	M2-3	25 March 2019	SRR24889203	20,082,478	19,467,632
	M3-1	01 April 2019	SRR24889202	23,973,758	23,122,643
	M3-2	01 April 2019	SRR24889201	17,751,557	14,385,246
	M3-3	01 April 2019	SRR24889200	16,939,100	15,343,827
	M4-1	07 April 2019	SRR24889199	25,393,019	23,710,871
	M4-2	07 April 2019	SRR24889233	22,442,744	20,910,442
	M4-3	07 April 2019	SRR24889232	27,606,319	26,204,746
	M5-1	13 April 2019	SRR24889231	28,887,298	27,099,908
	M5-2	13 April 2019	SRR24889230	22,201,888	21,349,940
	M5-3	13 April 2019	SRR24889229	24,331,541	23,006,465
Female cone	F1-1	19 March 2019	SRR24889228	24,660,578	23,260,555
	F1-2	19 March 2019	SRR24889227	23,231,253	22,130,832
	F2-1	25 March 2019	SRR24889226	21,536,523	20,786,185
	F2-2	25 March 2019	SRR24889225	17,000,533	16,119,565
	F3-1	01 April 2019	SRR24889224	22,713,496	19,223,728
	F3-2	01 April 2019	SRR24889222	16,588,232	14,210,260

	F4-1	07 April 2019	SRR24889221	27,437,779	25,595,867
	F4-2	07 April 2019	SRR24889220	34,396,437	32,163,913
	F5-1	13 April 2019	SRR24889219	22,727,525	20,877,478
	F5-2	13 April 2019	SRR24889218	27,931,253	26,386,624
Male structure of bisexual cone	BM4-1	07 April 2019	SRR24889210	22,603,820	21,087,741
	BM4-2	07 April 2019	SRR24889209	42,252,890	39,539,196
	BM4-3	07 April 2019	SRR24889208	26,408,979	24,899,933
	BM5-1	13 April 2019	SRR24889207	28,936,896	26,862,374
	BM5-2	13 April 2019	SRR24889206	20,626,480	19,609,321
	BM5-3	13 April 2019	SRR24889205	21,775,618	20,456,149
Female structure of bisexual cone	BF4-1	07 April 2019	SRR24889217	21,335,033	19,702,815
	BF4-2	07 April 2019	SRR24889216	19,951,803	18,648,417
	BF4-3	07 April 2019	SRR24889215	26,691,993	24,854,520
	BF5-1	13 April 2019	SRR24889214	27,709,738	26,471,791
	BF5-2	13 April 2019	SRR24889213	25,414,621	24,210,616
	BF5-3	13 April 2019	SRR24889211	24,280,681	22,969,324
