# Supplementary material for: Reciprocal expression of MADS-box genes and DNA methylation reconfiguration initiate bisexual cones in spruce
Source: Commun Biol. 2024 Jan 19;7:114. doi: 10.1038/s42003-024-05786-6 (PMC10799047; doi:10.1038/s42003-024-05786-6)
Supplement: Supplementary file 3 — Description of Additional Supplementary Files [file 42003_2024_5786_MOESM3_ESM.pdf]

Description of Additional Supplementary Files

File name: Supplementary Data 1
Description: All MADS-box genes used in phylogenetic reconstructions.

File name: Supplementary Data 2
Description: The body-methylated genes and their methylation levels in BF4.

File name: Supplementary Data 3
Description: The body-methylated genes and their methylation levels in BM4.

File name: Supplementary Data 4
Description: The body-methylated genes and their methylation levels in F4.

File name: Supplementary Data 5
Description: The body-methylated genes and their methylation levels in M4.

File name: Supplementary Data 6
Description: Genes with significant differences in both methylation levels and expression levels in M4 vs. F4.

File name: Supplementary Data 7
Description: Genes with significant differences in both methylation levels and expression levels in BM4 vs. BF4.

File name: Supplementary Data 8
Description: Annotation of genes with difference in both expression and methylation in M4 vs. F4 and BM4 vs. BF4.

File name: Supplementary Data 9
Description: HD-ZIP IV genes used in phylogenetic reconstruction.

File name: Supplementary Data 10
Description: All plant hormone contents.

File name: Supplementary Data 11
Description: Primers used in this study.

File name: Supplementary Data 12
Description: The numerical source data supporting both main and supplementary figures.
